# Combined effects of nitrogen fertilizer and biochar on the growth, yield, and quality of pepper

**DOI:** 10.1515/biol-2022-0882

**Published:** 2024-06-18

**Authors:** Chunyan Wu, Qiyuan Sun, Zeyue Ren, Nan Xia, Zhuang Wang, Hong Sun, Wei Wang

**Affiliations:** College of Horticulture, Jilin Agricultural University, Changchun, Jilin, 130118, China; College of Traditional Chinese Medicine, Jilin Agricultural Science and Technology University, Jilin, Jilin, 132001, China; Institute of Facility Agricultural Technology, Jilin Provincial Academy of Agricultural Machinery, Changchun, Jilin, 130022, China

**Keywords:** biochar, fertilizer, fruit quality, pepper yield, substrate properties

## Abstract

A pot experiment was conducted to investigate the combined effects of different nitrogen fertilizer levels (5, 25, and 45 kg of pure nitrogen per 667 m²) and biochar concentrations (0, 0.7, 1.4, and 2.1%) on the growth, yield, and fruit quality of pepper. The findings indicated that a combination of 25 kg/667 m^2^ of nitrogen and either 0.7% or 1.4% biochar significantly enhanced plant growth, yield, and fruit quality. Specifically, the N2 treatment (25 kg of pure nitrogen per 667 m²) increased substrate porosity, alkali-hydrolyzed nitrogen content, and available phosphorus content. It also boosted root activity and superoxide dismutase activity in pepper leaves, resulting in increased yield and better fruit quality. Furthermore, the proper addition of biochar (0.7–1.4% by weight) enhanced the physical and chemical properties of the substrate, including increased chlorophyll content and enzyme activity in plants, thereby leading to improved overall plant growth, yield, and fruit quality.

## 1 Introduction

Pepper, an annual or perennial herb of the genus *Capsicum* belonging to the family Solanaceae, is an herb native to the tropical regions of Central and South America [1]. Because of its rich nutrition, distinctive taste, and aroma, pepper plays a crucial role in human food, nutrition, and health [2,3]. In China, the annual cultivation area of pepper has remained stable at 2.1 million hm^2^, with a total output of 64 million tons, making it the largest planting area with the highest yield globally [4]. Nitrogen is crucial for plant growth and productivity, prompting farmers to utilize nitrogen fertilizer to enhance crop yield. However, plants can only absorb less than 50% of applied nitrogen, resulting in considerable nitrogen loss [5]. When combined with nitrogen fertilizer, biochar can mitigate nitrogen loss in the soil and enhance nitrogen retention in the soil [6].

The production and storage of biochar in soils have been considered as potential methods to reduce atmospheric CO_2_ concentration [7]. Biochar, produced through the oxygen-limited thermal decomposition of organic materials such as straw, wood waste, livestock, and poultry waste, is considered a sustainable biomass [8]. Its production process is cost-effective, sustainable, and easily scalable, attracting significant attention [9]. Biochar finds applications in enhancing soil physical and chemical properties, water purification, energy and gas storage, and agricultural productivity [10]. Additionally, it increases soil nutrient retention and water-holding capacity, facilitating improved plant root absorption while reducing the risk of water infiltration into rivers or underground reservoirs [11].

China, a significant agricultural country globally, produces a substantial amount of straw annually. Previous studies [12,13] showed that the total straw production of major crops in China reached 9.84 × 10^8^ tons in 2016. Maize, rice, and wheat accounted for 41.92, 23.23, and 18.36% of total straw production, respectively, making them the primary sources. Straw typically contains 31–41% crude fiber, 3–6% crude protein, and 42% nitrogen-free extract, with digestion energy ranging from 7.79 to 10.46 MJ/kg, making it a valuable biological resource [14]. The development, research, and utilization of biochar have become important measures to address waste resource utilization and the problems related to agriculture, rural areas, and farmers, significantly contributing to the efficient, ecological, and sustainable development of agriculture [15]. Although the appropriate addition of biochar can enhance the growth and yield of crops such as pepper [16], it is essential to note that biochar generally cannot replace fertilizers completely, as they may not provide all necessary nutrients [17,18].

To date, no attempts have been made to examine the combined effects of nitrogen fertilizers and biochar on pepper production. The following hypotheses were proposed: (i) the combined application of biochar and nitrogen fertilizers increased the yield of pepper by enhancing root activity, superoxide dismutase (SOD) activity, chlorophyll content, and enzyme activity. (ii) Different application conditions of biochar and nitrogen fertilizers had varying effects on the growth and production of pepper. The objectives of this study were twofold: (i) to determine the effects of different amounts of biochar and nitrogen fertilizers on the growth, fruit quality, and yield of potted peppers and (ii) to clarify the optimal application of biochar and nitrogen fertilizers for pepper plants, providing a theoretical basis for their application in pepper cultivation.

## 2 Materials and methods

### 2.1 Materials

Pepper seeds (*Capsicum annuum* L. cv. Jinfu 803) were sourced from Tianjin Chaoyan Seed and Seedling Technology Co., Ltd., Tianjin City, China. The seeds underwent evaluation and screening based on their appearance, size, weight, and shape to ensure their qualification for subsequent experiments. The commercial biochar used in the field experiment was derived from corn straw and produced by Liaoning Jinhefu Agricultural Technology Company, Liaoning Province, China. The nitrogen fertilizer tested was urea (*N* ≥ 46.4%), manufactured by Ordos New Energy Chemical Co., Ltd, Inner Mongolia, China. The physical and chemical properties of the substrate were as follows: alkali-hydrolyzable nitrogen 172.45 mg/kg, available phosphorus content 19.60 mg/kg, and available potassium content 65.18 mg/kg.

### 2.2 Apparatus

The instruments used in the experiment were as follows: 765UV-visible spectrophotometer, INESA Analytical Instrument Co., Ltd., Shanghai, China; TS-100 horizontal decolorization shaker, Haimen Kylin-Bell Lab Instruments Co., Ltd., Haimen, China; F96PRO fluorescence spectrophotometer, Shanghai Lengguang Technology Co., Ltd., Shanghai, China; TDL-80-2B low-speed centrifuge, Shanghai Yitian Scientific Instrument Co., Ltd., Shanghai, China; and HH-S2 digital thermostatic water bath, Jintan Medical Instrument Factory Co., Ltd., Changzhou, China.

### 2.3 Experimental setup

The experiment was conducted at the Facility Agriculture Science and Engineering Base and Horticulture Comprehensive Laboratory of the College of Horticulture, Jilin Agricultural University, China. The pot treatments comprised three nitrogen levels: pure nitrogen 5 kg/667 m^2^ (N1), 25 kg/667 m^2^ (N2), and 45 kg/667 m^2^ (N3) and four biochar rates: 0% (B0), 0.7% (B1), 1.4% (B2), and 2.1% (B3) by weight, resulting in 12 treatments. A randomized block design was used in the experiment, with each treatment replicated three times. The setting of pure nitrogen concentrations allowed for a clearer observation of the impact of biochar on the substrate and pepper quality and yield.

Pepper seeds were sown in a solar greenhouse on March 15, 2017. When the seedlings reached the two-leaf stage, they were transplanted into 10 cm × 10 cm nutrient bowls. The nutrient soil allocation and seedling stage management followed local conventional practices, with a peat-to-vermiculite ratio of 2:1 for the nutrient soil mix to maintain a substrate moisture content of 70–80%. The seedling temperature was managed according to appropriate growth conditions. Pepper seedlings were then transferred to pots filled with a garden soil–turf mixture (4:1, *v*/*v*), with each pot containing 12 kg of substrate thoroughly mixed with 1% chicken manure (nitrogen content 6.68 g/kg) and appropriate biochar content according to the treatments. Pepper seedlings were transplanted with a spacing of 40 cm along a row and 60 cm between rows. Nitrogen fertilizer was applied according to the treatments, with recommended doses of P and K (15 and 25 kg/667 m^2^, respectively) applied as diammonium phosphate and potassium sulfate, respectively.

The fruits of the peppers tested matured on July 19 and were harvested successfully. Mature fruits were selected for yield and quality determination.

### 2.4 Substrate measurements

Substrate bulk density was assessed using the cutting-ring method [19]. Substrate porosity was calculated as follows [19]:

\[\text{Porosity}=\text{(1}-\text{substrate}\hspace{.25em}\text{bulk}\hspace{.25em}\text{density}/\text{substrate}\hspace{.25em}\text{specific}\hspace{.25em}\text{gravity)}\times 100 \% .]\]

The content of available phosphorus was determined using the 0.5 M NaHCO_3_ method, available nitrogen content was analyzed using the alkali-hydrolyzing nitrogen method, and available potassium content in the substrate was measured using the flame photometry method [19].

### 2.5 Measurements of growth and physiological indexes

The plant height was measured using a ruler, whereas the stem diameter was measured with a Vernier caliper. The entire shoot biomass was harvested by cutting the shoots on the surface of the substrate, and the fresh weight was recorded. The roots were then separated from the soil and weighed. Subsequently, both the shoots and roots were oven-dried at 70°C until a constant dry weight was achieved.

The chlorophyll content was determined using the spectrophotometric method, and the root activity was assessed using the TTC method. The SOD activity was determined using the photochemical reduction of nitroblue tetrazolium method, and the peroxidase (POD) activity was measured using the guaiacol method [20].

### 2.6 Fruit yield and quality assessment

At maturity, 10 ripe pepper fruits were randomly selected from each treatment for yield determination and quality analysis. The soluble sugar content (SS) was measured using the anthrone method, the soluble protein content (SP) was determined by the Coomassie Bright Blue G-250 staining method, the vitamin C content (VC) was assessed by molybdenum blue colorimetry, the organic acid content (OA) was determined by alkaline titration, and the free amino acid content (FAA) was measured using ninhydrin coloration method [20].

### 2.7 Statistical analysis

All data were analyzed using two-way analysis of variance (ANOVA), and the means were compared using the least significant difference (LSD) test. The significance between treatments was evaluated at a probability of 0.05. All statistical analyses were performed using DPS software.

## 3 Results

### 3.1 Physical and chemical properties of substrate

**Figure 1:**
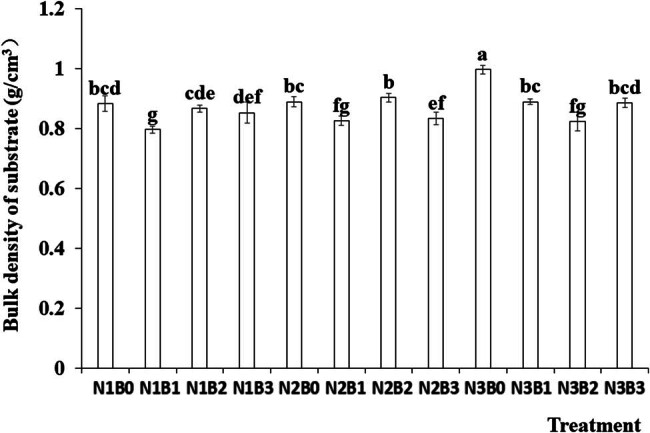
Effects of nitrogen fertilizer and biochar on the bulk density of the substrate. Note: B, Biochar amendments (0, 0.7, 1.4, and 2.1% by weight); N, nitrogen treatments (N1, N2, and N3 treatments of 5, 25, and 45 kg/667 m^2^, respectively). Error bars represent standard error (*n* = 3). Different letters above the bars within each panel indicate significant differences between treatments (*P* ≤ 0.05).

Substrate bulk density plays a critical role in various biochemical processes such as water movement and salt migration by influencing the water-to-gas ratio, which is an indicator of substrate fertility. As shown in Figure 1, the bulk density of the substrate decreased under all biochar treatments compared with no-biochar treatment. Nitrogen fertilizer did not significantly affect substrate bulk density. The highest substrate bulk density was observed under the combined treatment of 45 kg/667 m^2^ nitrogen fertilizer and no biochar (N3B0), whereas the lowest value was recorded under the treatment with 5 kg/667 m^2^ nitrogen fertilizer and 0.7% biochar amendment (N1B1).

**Figure 2:**
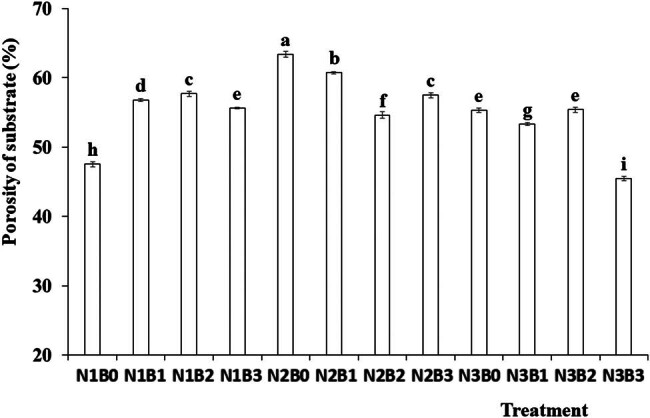
Effects of nitrogen fertilizer and biochar on porosity of the substrate. Note: B, biochar amendments (0, 0.7, 1.4, and 2.1% by weight); N, nitrogen treatments (N1, N2, and N3 treatments of 5, 25, 45 kg/667 m^2^, respectively). Error bars represent standard error (*n* = 3). Different letters above the bars within each panel indicate significant differences between treatments (*P* ≤ 0.05).

Figure 2 shows the mean values of substrate porosity under three nitrogen fertilizer and four biochar treatments. The substrate porosity values were significantly affected by both nitrogen fertilizer and biochar treatments. The N2 treatment exhibited the highest porosity, surpassing that under the N1 and N3 treatments. Moreover, the B1 and B2 biochar amendments significantly increased porosity compared with no-biochar treatment. The highest porosity value was recorded under the N2B0 treatment, which was significantly higher than under the other treatments.

**Figure 3:**
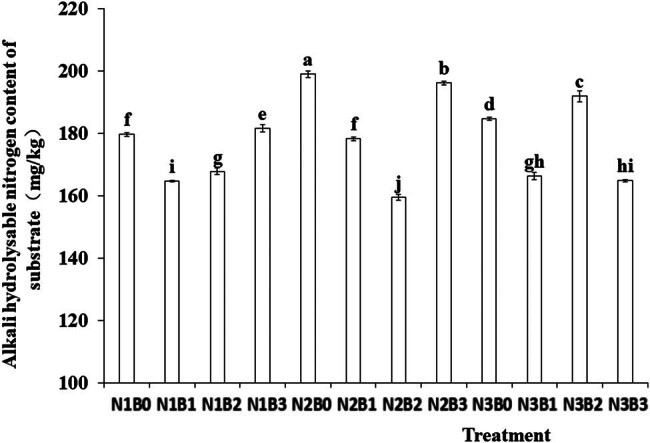
Effects of nitrogen fertilizer and biochar combination on base-hydrolyzed nitrogen. Note: B, Biochar amendments (0, 0.7, 1.4, and 2.1% by weight); N, nitrogen treatments (N1, N2, and N3 treatments of 5, 25, and 45 kg/667 m^2^, respectively). Error bars represent standard error (*n* = 3). Different letters above the bars within each panel indicate significant differences between treatments (*P* ≤ 0.05).

Alkali-hydrolyzed nitrogen is a crucial indicator reflecting soil nitrogen supply capacity, including both inorganic and organic nitrogen. Its content is closely associated with organic matter content and is unstable in soil. Both nitrogen levels and biochar significantly affected the alkali-hydrolyzed nitrogen content in the substrate (Figure 3). The N2 treatment exhibited comparatively higher alkali-hydrolyzed nitrogen content than N1 and N3 N2 treatments. The highest alkali-hydrolyzed nitrogen content was 187.76 mg/kg under B0 treatment. The highest alkali-hydrolyzed nitrogen content was 198.95 mg/kg under the N2B0 treatment, whereas the lowest content of alkali-hydrolyzed nitrogen was observed under the N2B2 treatment.

**Figure 4:**
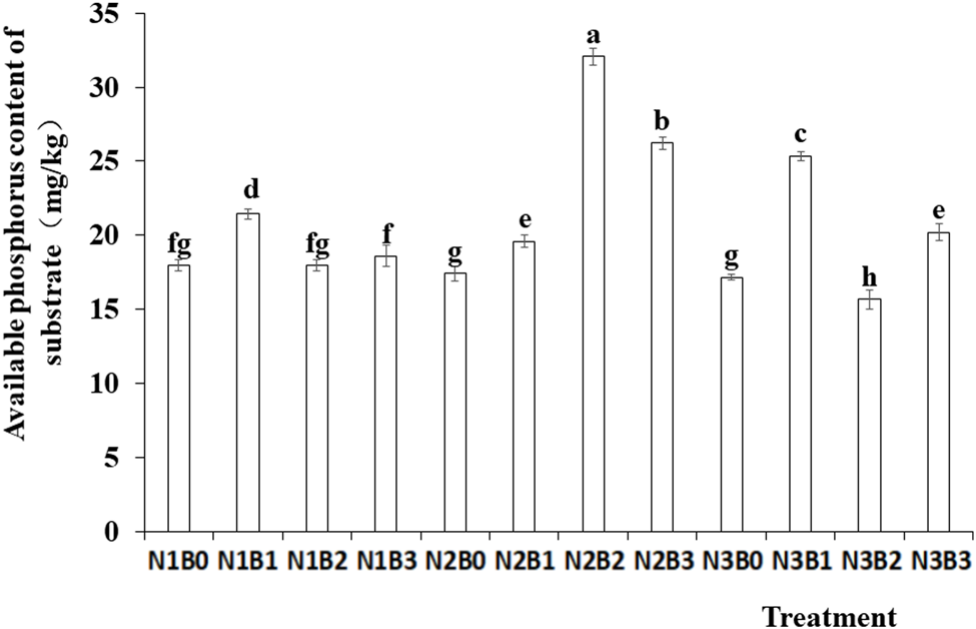
Effects of nitrogen fertilizer and biochar combination on available phosphorus content in the substrate. Note: B, Biochar amendments (0, 0.7, 1.4, and 2.1% by weight); N, nitrogen treatments (N1, N2, and N3 treatments of 5, 25, and 45 kg/667 m^2^, respectively). Error bars represent standard error (*n* = 3). Different letters above the bars within each panel indicate significant differences between treatments (*P* ≤ 0.05).

The impact of nitrogen fertilizer and biochar on available phosphorus content in the substrate is depicted in Figure 4. The data indicated a significant effect of different nitrogen levels on available phosphorus content. The highest available phosphorus content was 23.83 mg/kg in the N2 treatment. Furthermore, biochar amendment significantly increased available phosphorus content compared with no-biochar control. The highest available phosphorus content was observed under the N2B2 treatment.

**Figure 5:**
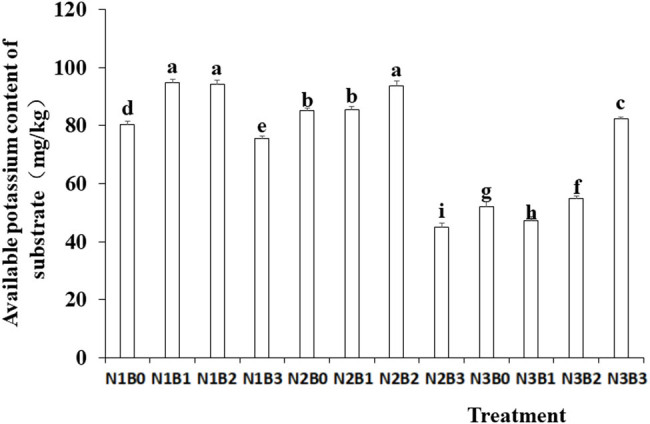
Effects of nitrogen fertilizer and biochar combination on available potassium content in the substrate. Note: B, Biochar amendments (0, 0.7, 1.4, and 2.1% by weight); N, nitrogen treatments (N1, N2, and N3 treatments of 5, 25, and 45 kg/667 m^2^, respectively). Error bars represent standard error (*n* = 3). Different letters above the bars within each panel indicate significant differences between treatments (*P* ≤ 0.05).

Nitrogen fertilizer and biochar treatments exhibited significant effects on available potassium content (Figure 5). The available potassium content in the substrate significantly decreased with the increase in the amount of nitrogen fertilizer applied. The available potassium content under the B1 and B2 treatments was higher than under no-biochar control. The highest available potassium content was observed under the N1B1 treatment, followed by the N1B2 and N2B2 treatments. No significant difference was observed among the three treatments.

### 3.2 Growth parameters

**Figure 6:**
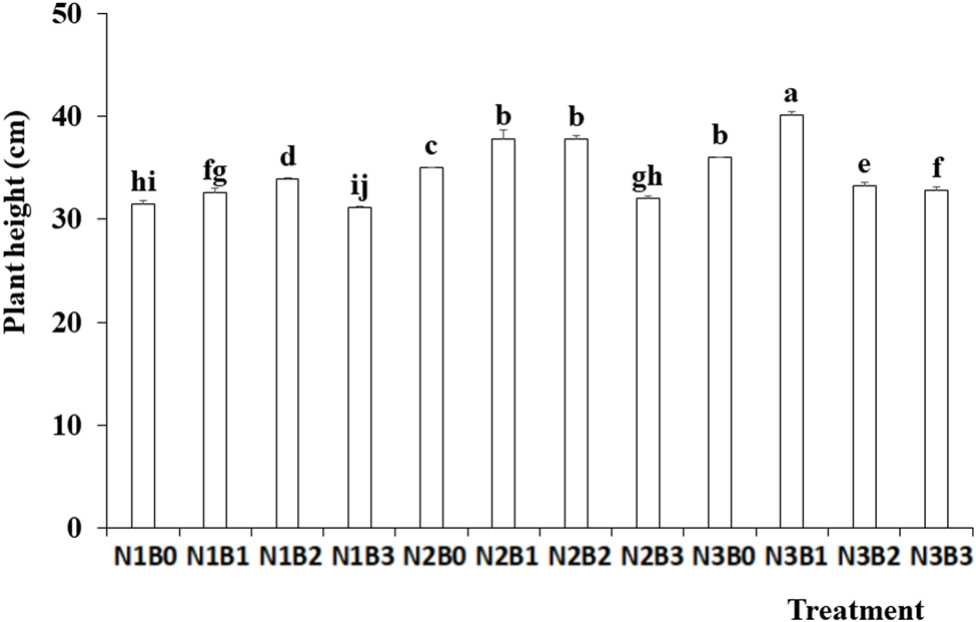
Effects of nitrogen fertilizer and biochar combination on the plant height of pepper. Note: B, Biochar amendments (0, 0.7, 1.4, and 2.1% by weight); N, nitrogen treatments (N1, N2, and N3 treatments of 5, 25, and 45 kg/667 m^2^, respectively). Error bars represent standard error (*n* = 3). Different letters above the bars within each panel indicate significant differences between treatments (*P* ≤ 0.05).

Plant height was significantly affected by both nitrogen fertilizer and biochar treatments (Figure 6). The maximum plant height was observed under the N2 treatment, followed by the N3 treatment. No significant difference was observed between the two fertilizer treatments. The plant height increased significantly under the B1 and B2 treatments compared with the control and B3 treatments. The greatest plant height was 40.13 cm under the N3B1 treatment, which was significantly greater than that under the other treatments.

**Figure 7:**
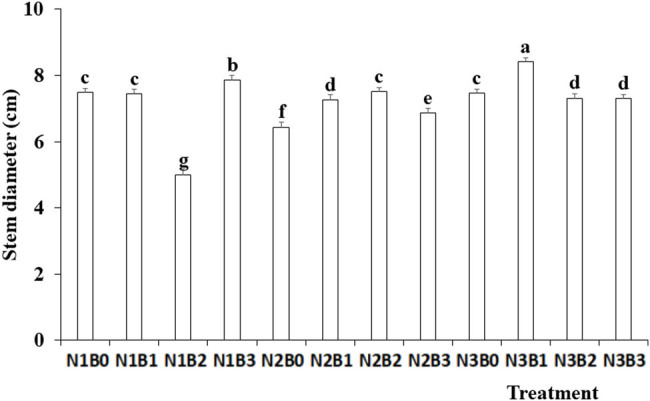
Effects of nitrogen fertilizer and biochar combination on the stem diameter of pepper. Note: B, Biochar amendments (0, 0.7, 1.4, and 2.1% by weight); N, nitrogen treatments (N1, N2, and N3 treatments of 5, 25, and 45 kg/667 m^2^, respectively). Error bars represent standard error (*n* = 3). Different letters above the bars within each panel indicate significant differences between treatments (*P* ≤ 0.05).

Both nitrogen fertilizer and biochar treatment had a significant effect on stem diameter (Figure 7). It increased with the increase in nitrogen fertilizer application. Biochar amendment also increased the stem diameter compared with the no-biochar control. Among the 12 treatments, the maximum stem diameter occurred under N3B1 treatment.

Fresh plant weight (FPW) increased significantly in plants treated with biochar (Table 1). Nitrogen fertilizer also had a significant effect on FPW, with the greatest FPW under N2 treatment. The maximum FPW was observed under N2B1 treatment. Both nitrogen fertilizer and biochar had a significant effect on fresh root weight (FRW). The greatest FRW was found under N3B1 treatment compared with other treatments.

**Table 1:** Effects of nitrogen fertilizer and biochar on the plant growth parameters of pepper

Treatment	FPW (g)	FRW (g)	DPW (g)	DRW (g)
N1B0	219.98 ± 0.70c	27.44 ± 0.10e	34.08 ± 0.32c	5.58 ± 0.04b
N1B1	215.89 ± 1.03e	38.23 ± 0.18a	33.07 ± 0.15d	4.85 ± 0.22d
N1B2	221.06 ± 0.37c	32.60 ± 0.12c	34.83 ± 0.79bc	5.52 ± 0.30bc
N1B3	204.46 ± 0.26h	25.56 ± 0.25f	30.35 ± 0.81gh	5.41 ± 0.06bc
N2B0	209.20 ± 0.42g	22.45 ± 0.37g	32.49 ± 0.49de	6.29 ± 0.07a
N2B1	250.91 ± 1.34a	22.62 ± 0.39g	31.77 ± 0.24ef	5.37 ± 0.22bc
N2B2	210.76 ± 0.46f	28.41 ± 0.15d	35.29 ± 0.44b	6.45 ± 0.24a
N2B3	216.57 ± 1.15de	21.49 ± 0.17h	29.62 ± 0.41h	5.49 ± 0.07bc
N3B0	192.02 ± 1.21j	27.28 ± 0.07e	31.56 ± 0.36ef	5.12 ± 0.17cd
N3B1	230.96 ± 0.68b	35.58 ± 0.11b	36.61 ± 0.23a	5.57 ± 0.39b
N3B2	193.51 ± 0.66i	20.69 ± 0.07i	31.02 ± 1.08fg	5.52 ± 0.41bc
N3B3	217.42 ± 0.66d	25.27 ± 0.18f	32.54 ± 0.29de	5.31 ± 0.29bc
ANOVA				
N	**	**	**	**
B	**	**	**	**
N × B	**	**	**	**

Note: DPW, dry plant weight; DRW, dry root weight; FPW, fresh plant weight; FRW, fresh root weight. The data are the means of three replicates. Values within the same columns followed by different letters are significantly different at *P* < 0.05 as per the LSD test. B, biochar amendments (0, 0.7, 1.4, and 2.1% by weight); N, nitrogen treatments (N1, N2, and N3 treatments of 5, 25, and 45 kg/667 m^2^, respectively). Means followed by the same lowercase letter in each column and the same uppercase letter in each row do not differ at the 0.05 probability level according to Duncan’s test. ** *P* < 0.01.

Dry plant weight (DPW) and dry root weight (DRW) were significantly affected by both nitrogen fertilizer and biochar treatments (Table 1). DPW was significantly higher under the N1 and N3 treatments compared with the N2 treatment. The B1 and B2 treatments significantly increased DPW compared with the control. Among the combination treatments, N3B2 resulted in the highest DPW, whereas the smallest value was observed under the N2B3 treatment. The largest increases in DRW were observed under the N2B2 and N2B0 treatments.

Nitrogen fertilizer and biochar had a significant impact on chlorophyll content. The chlorophyll content significantly increased with higher nitrogen fertilizer levels, and the B1 treatment significantly increased chlorophyll content compared with the control. The N3B1 treatment led to the highest chlorophyll content.

Table 2 illustrates the significant effect of nitrogen fertilizer and biochar on root activity. The root activity increased significantly on increasing nitrogen fertilizer levels from N1 to N3; however, no significant increase was observed under N2 and N3 treatments. Additionally, biochar amendments significantly increased root activity compared with the control, with the highest root activity observed under the N2B2 combination treatment.

**Table 2:** Effects of nitrogen fertilizer and biochar combination on the physiological index of pepper leaves

Treatment	Chlorophyll content (mg/g)	Root activity (µg/g h)	POD (µ/g)	SOD (µ/g)
N1B0	3.01 ± 0.15h	161.33 ± 0.92i	524.00 ± 1.73k	211.04 ± 0.67hi
N1B1	3.00 ± 0.22h	182.67 ± 0.15d	625.00 ± 0.00g	213.74 ± 0.33g
N1B2	2.07 ± 0.56j	177.33 ± 0.49e	691.33 ± 2.31b	226.37 ± 0.47f
N1B3	2.44 ± 0.34i	190.00 ± 0.67b	630.00 ± 5.00g	242.53 ± 0.04c
N2B0	3.54 ± 0.24f	171.33 ± 0.29g	610.00 ± 5.00h	247.31 ± 2.14a
N2B1	3.7 ± 0.15e	187.67 ± 0.34c	647.33 ± 2.52e	240.92 ± 0.25d
N2B2	2.47 ± 0.53i	192.67 ± 0.19a	636.67 ± 2.89f	230.11 ± 0.46e
N2B3	3.75 ± 0.22d	175.67 ± 0.47f	553.33 ± 5.77j	245.86 ± 0.18b
N3B0	4.37 ± 0.19c	181.67 ± 0.36d	678.33 ± 2.89c	231.09 ± 0.12e
N3B1	4.61 ± 0.16a	192.67 ± 0.53a	655.00 ± 5.00d	209.89 ± 0.28i
N3B2	4.46 ± 0.39b	186.33 ± 0.30c	778.33 ± 2.89a	211.98 ± 0.50h
N3B3	3.44 ± 0.16g	169.33 ± 0.51h	563.33 ± 2.89i	225.19 ± 0.08f
ANOVA				
N	**	**	**	**
B	**	**	**	**
N × B	**	**	**	**

Note: B, biochar amendments (0, 0.7, 1.4, and 2.1% by weight); N, nitrogen treatments (N1, N2, and N3 treatments of 5, 25, and 45 kg/667 m^2^, respectively). Means followed by the same lowercase letter in each column and the same uppercase letter in each row do not differ at 0.05 probability level by the Duncan’s test. ** *P* < 0.01.

The influence of nitrogen fertilizer and biochar on POD activity is presented in Table 2. Both nitrogen fertilizer and biochar had significant effects on POD activity, with the highest activity observed under the N3 treatment. Moreover, B1 and B2 treatments increased POD activity compared with the control, with the maximum activity recorded under the N3B2 treatment which was significantly greater than that under other treatments.

Nitrogen fertilizer and biochar both exhibited significant effects on SOD activity. The highest SOD activity was observed under the N2 treatment. A significant increase in the SOD activity was observed under the B3 treatment compared with B0 treatment. The highest SOD activity was recorded under the N2B0 treatment.

### 3.3 Yield

**Figure 8:**
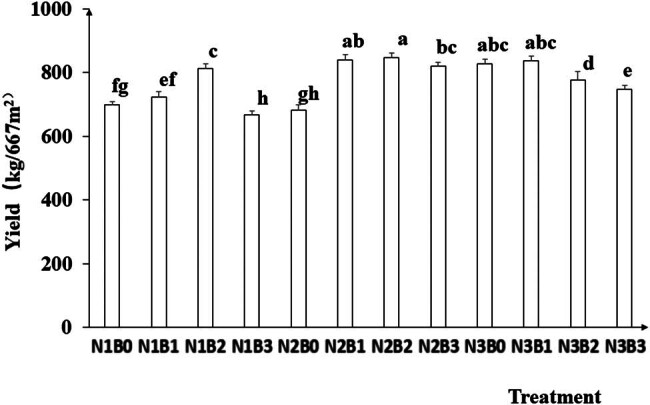
Effects of nitrogen fertilizer and biochar combination on the yield of pepper. Note: B, Biochar amendments (0, 0.7, 1.4, and 2.1% by weight); N, nitrogen treatments (N1, N2, and N3 treatments of 5, 25, and 45 kg/667 m^2^, respectively). Error bars represent standard error (*n* = 3). Different letters above the bars within each panel indicate significant differences between treatments (*P* ≤ 0.05).

Yield was significantly affected by nitrogen fertilizer and biochar treatments (Figure 8). The yield in the N2 treatment was higher than the N3 and N1 treatments, and biochar amendment increased the yield compared with non-biochar control. The maximum yield was recorded in the N2B2 treatment, followed by the N2B1 and N3B1 treatment. No significant difference was observed among the three treatments.

### 3.4 Fruit quality

As shown in Table 3, nitrogen fertilizer and biochar treatments exhibited significant effects on the SS content. The SS content increased with the increase in the nitrogen fertilizer level. The SS content was higher under B1 and B2 treatments compared with no-biochar control. The SS content under the N2B1 treatment was 2.27%, followed by the N3B1 and N2B1 treatments, which was 2.25% and 2.20%, respectively. No significant difference was observed among the three treatments.

**Table 3:** Effects of nitrogen fertilizer and biochar on pepper fruit quality

Treatment	SS (mg/g)	SP (mg/g)	OA (%)	VC (mg/100 g)	FAA (µg/100 g)
N1B0	1.74 ± 0.08cd	15.90 ± 0.67f	0.23 ± 0.03def	40.38 ± 1.14f	632.75 ± 3.45de
N1B1	1.54 ± 0.08de	22.07 ± 0.27d	0.26 ± 0.04cde	42.45 ± 1.36e	629.43 ± 4.35de
N1B2	1.80 ± 0.11c	18.87 ± 0.57e	0.20 ± 0.03ef	47.06 ± 0.66c	637.08 ± 7.98cd
N1B3	1.44 ± 0.20e	21.40 ± 0.64d	0.25 ± 0.03cde	42.45 ± 0.24e	638.87 ± 9.77cd
N2B0	2.12 ± 0.10ab	19.23 ± 0.61e	0.20 ± 0.02ef	47.21 ± 0.54bc	635.30 ± 5.74d
N2B1	2.20 ± 0.03ab	33.10 ± 0.55ab	0.29 ± 0.04c	51.72 ± 1.32a	611.83 ± 4.21e
N2B2	2.27 ± 0.29a	31.93 ± 0.35c	0.36 ± 0.02a	44.53 ± 1.61d	638.10 ± 3.06cd
N2B3	1.97 ± 0.08bc	32.07 ± 0.72bc	0.37 ± 0.03b	51.21 ± 1.45a	658.76 ± 9.28bc
N3B0	2.12 ± 0.15ab	33.07 ± 1.16ab	0.25 ± 0.04cde	48.90 ± 1.34b	645.75 ± 10.95bcd
N3B1	2.25 ± 0.12a	33.20 ± 0.84a	0.22 ± 0.04def	51.84 ± 0.76a	665.39 ± 2.60ab
N3B2	2.14 ± 0.14ab	19.63 ± 0.75e	0.27 ± 0.02cd	43.14 ± 0.67de	658.25 ± 10.44bc
N3B3	2.13 ± 0.12ab	31.57 ± 0.76c	0.28 ± 0.02c	47.98 ± 0.61bc	684.27 ± 8.04a

Note: B, biochar amendments (0, 0.7, 1.4, and 2.1% by weight); FAA, free amino acid content; N, nitrogen treatments (N1, N2, and N3 treatments of 5, 25, and 45 kg/667 m^2^, respectively); OA, organic acid content; SP, soluble protein content; SS, soluble sugar content; VC, vitamin C content;. The data are represented as means of three replicates. Different letters within the same column indicate significant differences at *P* < 0.05 as revealed by the LSD test.

The SP content significantly increased with an increase in nitrogen fertilizer level (Table 3). Biochar amendment also increased SP content compared with the no-biochar treatment. The highest SP values were observed under N3B1, N2B1, and N3B0 treatments.

The composition and content of OAs are crucial factors affecting fruit quality. The measured OA values are presented in Table 3. The OA content under N2 treatment was significantly higher than that under N3 and N1 treatments. It increased significantly with an increase in BA. The highest OA values were recorded under the N2B2 and N2B3 treatments, with no significant difference between the two treatments. The lowest OA content was observed under the N1B2 and N2B0 treatments.

VC is an important antioxidant and a key factor representing fruit nutritional quality [21]. The effects of nitrogen fertilizer and biochar on the VC content of pepper fruits were significant (Table 3). The VC content under N2 and N3 treatments was significantly higher than that under N1 treatment. The VC content under the B1 and B3 treatments was significantly higher than that under the B0 and B2 treatments. The highest VC content was observed under the N3B1, N2B1, and N2B3 treatments, with no significant difference among the three treatments. The lowest VC content was found under the N1B1 and N1B0 treatments.

The content of FAAs was significantly affected by nitrogen fertilizer and biochar treatments (Table 3). The FAA content increased with an increase in nitrogen fertilizer application, but no significant difference was observed between N2 and N1 treatments. The FAA content under the B3 treatment was significantly higher than that under the other treatments. The highest FAA content was observed under the N3B3 and N3B1 treatments.

### 3.5 Principal component analysis

Single-index analysis may not fully capture the effects of biochar and nitrogen fertilizer on the quality and production of pepper.

To comprehensively evaluate the impact of different treatments on pepper quality, it is essential to assess various treatments in combination with each quality index. This approach allows for determining the contribution of each quality index to overall pepper quality, thereby facilitating a better understanding of which biochar contents and nitrogen fertilizer concentrations are most beneficial for producing high-quality pepper.

The principal component analysis was used to comprehensively analyze six quality-related indicators, such as yield, quality, aboveground dry weight, underground dry weight, aboveground fresh weight, and underground fresh weight, to evaluate the most effective biochar and nitrogen fertilizer treatment.

Initially, the data were dimensionally reduced to obtain standardized data. Subsequently, the standardized data were analyzed to determine the eigenvalues, eigenvectors, and cumulative contribution rate of the correlation matrix (Table 4). The principal components with eigenvalues >1 were selected for further analysis.

**Table 4:** Eigenvalues and variance contribution rates of principal component analysis

Component	Initial eigenvalues			Sum of squared initial loading factors		
	Eigenvalue	Contribution rate (%)	Cumulative contribution rate (%)	Eigenvalue	Contribution rate (%)	Cumulative contribution rate (%)
1	3.479	34.791	34.791	3.479	34.791	34.791
2	2.087	20.868	55.659	2.087	20.868	55.659
3	1.433	14.328	69.987	1.433	14.328	69.987
4	1.209	12.089	82.077	1.209	12.089	82.077
5	0.819	8.186	90.262	/	/	/
6	0.498	4.976	95.239	/	/	/
7	0.248	2.485	97.724	/	/	/
8	0.173	1.734	99.458	/	/	/
9	0.051	0.508	99.965	/	/	/
10	0.003	0.035	100	/	/	/

The relationship between each quality index and the first four principal components was calculated as follows:

First principal component:

(1) \[\begin{array}{l}{F}_{1}=0.1968\times {X}_{1}\hspace{.5em}\mbox{--}\hspace{.5em}0.0912\times {X}_{2}+0.0686\times {X}_{3}+0.104\hspace{2em}\times {X}_{4}+0.4542\times {X}_{5}+0.4429\times {X}_{6}+0.4778\hspace{2em}\times {X}_{7}+0.2901\times {X}_{8}+0.4456\times {X}_{9}+0.155\times {X}_{10}.\end{array}]\]

Second principal component:

(2) \[\begin{array}{l}{F}_{2}=0.3738\times {X}_{1}+0.5822\times {X}_{2}+0.5939\times {X}_{3}\hspace{.5em}\mbox{--}\hspace{.5em}0.0443\times {X}_{4}+0.1384\times {X}_{5}\hspace{.5em}\mbox{--}\hspace{.5em}0.0277\times {X}_{6}\hspace{.5em}\mbox{--}\hspace{.5em}0.018\times {X}_{7}\hspace{.5em}\mbox{--}\hspace{.5em}0.3337\times {X}_{8}+0.0581\times {X}_{9}\hspace{.5em}\mbox{--}\hspace{.5em}0.1779\times {X}_{10}.\end{array}]\]

Third principal component:

(3) \[\begin{array}{l}{F}_{3}=(\mbox{--}0.1487)\times {X}_{1}\mbox{--}0.1905\times {X}_{2}+0.3417\times {X}_{3}+0.7794\times {X}_{4}\hspace{.5em}\mbox{--}\hspace{.5em}0.0635\times {X}_{5}+0.345\times {X}_{6}\hspace{.5em}\mbox{--}\hspace{.5em}0.2289\times {X}_{7}\hspace{.5em}\mbox{--}\hspace{.5em}0.0485\times {X}_{8}\hspace{.5em}\mbox{--}\hspace{.5em}0.1946\times {X}_{9}\hspace{2em}\mbox{--}\hspace{.5em}0.0409\times {X}_{10}.\end{array}]\]

Fourth principal component:

(4) \[\begin{array}{l}{F}_{4}=(\mbox{--}0.5103)\times {X}_{1}+0.2729\times {X}_{2}+0.2065\times {X}_{3}\hspace{.5em}\mbox{--}\hspace{.5em}0.1264\times {X}_{4}+0.0018\times {X}_{5}+0.0937\times {X}_{6}+0.07\times {X}_{7}\hspace{.5em}\mbox{--}\hspace{.5em}0.1364\times {X}_{8}\mbox{--}\text{}0.0646\times {X}_{9}+0.755\times {X}_{10}.\end{array}]\]

In the formulas, *F*_1_, *F*_2_, *F*_3_, and *F*_4_ correspond to the scores of the first, second, third, and fourth principal components, respectively. *X*_1_, *X*_2_, *X*_3_, *X*_4_, *X*_5_, *X*_6_, *X*_7_, *X*_8_, *X*_9_, and *X*_10_ represent the aboveground fresh weight, underground fresh weight, aboveground dry weight, underground dry weight, yield, SS, SP, OA, VC, and FAA after eliminating the dimensional relationship between variables.

The comprehensive evaluation function utilizes the variance contribution rate corresponding to each principal component as the weight:

(5) \[\text{Comprehensive}\hspace{.25em}\text{score}\hspace{.25em}F=0.4239\times {F}_{1}+0.2542\times {F}_{2}+0.1746\times {F}_{3}+0.1473\times {F}_{4}.]\]

The standardized values were input into equations (1)–(4), and then the calculated values were applied into equation (5). Finally, the comprehensive evaluation of pepper quality indexes under biochar and nitrogen fertilizer treatments was conducted (Table 5).

**Table 5:** Comprehensive assessment of pepper quality under different nitrogen fertilizer levels and biochar concentrations

Component	*F*_1_	*F*_2_	*F*_3_	*F*_4_	*F*	Ranking
N1B0	–2.56	0.66	0.75	–0.51	–0.86	10
N1B1	–2.35	1.45	–1.74	0.04	–0.93	11
N1B2	–0.84	1.95	0.09	0.01	0.16	6
N1B3	–2.82	–1.21	–0.67	–0.30	–1.67	12
N2B0	–1.06	–0.54	2.12	–0.55	–0.30	8
N2B1	2.29	0.44	–0.90	–2.83	0.51	3
N2B2	1.90	0.20	2.25	–0.12	1.23	2
N2B3	2.00	–2.18	–1.06	–0.36	0.06	7
N3B0	0.86	–0.65	–0.97	0.96	0.17	5
N3B1	2.18	2.64	0.03	1.32	1.80	1
N3B2	–0.65	–2.00	0.66	0.80	–0.55	9
N3B3	1.06	–0.76	–0.56	1.54	0.38	4

Note: B, Biochar amendments (0, 0.7, 1.4, and 2.1% by weight), N, nitrogen treatments (N1, N2, and N3 treatments for 5, 25, and 45 kg/667 m^2^); *F*_1_, first principal component; *F*_2_, second principal component; *F*_3_, third principal component; *F*_4_, fourth principal component; *F*, comprehensive score.

The comprehensive evaluation indicated that the treatment ranked N3B1 as the first, with a score of 1.80, followed by N2B2, which scored 1.23, resulting in a difference of 0.57. The remaining eight groups of treatments were as follows, according to the comprehensive evaluation results: N2B1, N3B3, N3B0, N1B2, N2B3, N2B0, N3B2, N1B0, N1B1, and N1B3, with scores of 0.51, 0.38, 0.17, 0.16, 0.06, –0.30, –0.55, –0.86, –0.93, and –1.67, respectively.

## 4 Discussion

The rational application of biochar and fertilizer is vital for efficiently utilizing agricultural straw resources to reduce agricultural pollution, enhance fertilizer utilization rates, boost crop yields, and maintain soil moisture while reducing nutrient loss. The pore structure of biochar can augment soil porosity, decrease soil bulk density, and improve soil texture and tillage performance [22,23]. Additionally, biochar incorporation enhances irrigation water efficiency and influences plant growth by improving the matrix medium [24]. The findings of the present study revealed that biochar significantly reduced substrate bulk density and increased substrate porosity, aligning with previous studies demonstrating a decrease in soil bulk density with increased peat content [25,26]. Besides lower bulk density, adding biochar to the substrate led to even lower densities compared with conventional substrates, potentially due to the use of garden soil and peat as the cultivation substrate.

Biochar serves to restore soil fertility and enhance soil productivity through various mechanisms. First, biochar contains mineral nutrients like phosphorus, potassium, calcium, magnesium, and nitrogen, enriching soil nutrient levels [27]. Second, its porous structure and large surface area, along with substantial negative surface, enable it to absorb more water and nutrient ions, facilitating nutrient uptake by plants [28]. Third, biochar’s ability to restrain and retain soil nutrients reduces leaching and erosion losses, thereby enhancing soil stability and organic carbon content, ultimately boosting total crop yield [29]. Fourth, biochar fosters an optimal environment for soil microorganisms [30], facilitating nutrient cycling in soil ecosystems and contributing to soil quality and health maintenance [31]. Although biochar itself contains relatively low mineral element content, its effects on soil nutrients are primarily mediated by its properties and indirect impacts on soil physicochemical properties and microorganisms [32]. The present study found that biochar treatments significantly increased available phosphorus content compared with non-biochar treatments, with the highest available potassium content observed in the B2 treatment. Alkali-hydrolyzed nitrogen and available phosphorus content peaked under medium nitrogen application, whereas the available potassium content decreased significantly with the increase in nitrogen application.

The biochar application as a soil amendment holds promise for promoting growth and enhancing crop productivity [33,34]. Previous studies demonstrated biochar’s growth-promoting effects on various crops like tomatoes [21], peppers, lettuce [35], beans [36], potatoes [37], cowpeas, and radishes [38,39]. However, its impact varies depending on biochar type, application rate, and crop species. For instance, biochar with high volatility may hinder nitrogen absorption and crop growth [40,41]. However, different biochar extracts can have distinct effects on seed germination. Moreover, the sludge biochar and cow dung biochar extracts showed a more obvious inhibitory effect on the germination of pepper seeds [42]. Additionally, the application amount of biochar is also a crucial factor. Biochar application has a specific range of effectiveness [43]. Although an appropriate amount of biochar can promote pepper growth and development, higher biochar additions do not significantly promote pepper growth [44]. For example, in soils with a lower available nutrients or nitrogen, low amount of biochar is likely to promote crop growth and increase crop yield, whereas a higher amount of biochar can reduce growth and yield [43,45]. The impact of biochar varies depending upon the type of crop. Even under identical conditions, the effects of applying the same biochar to different crops can differ in terms of growth and yield [46].

The effect of biochar for soil improvement is closely related to fertilizer management. Kebede et al. found that the combined application of biochar and compost significantly affected pH, OC, TN, P, K, and other soil characteristics and pepper growth [16]. However, the impact of biochar on pepper yield is not solely determined by the amount applied [47]. Liu et al. conducted field cylindrical tube cultivation experiments on soybean, and the results showed that the combination of biochar and nitrogen fertilizer mainly affected the yield by adjusting the number of grains per plant [48]. However, biochar inhibited the accumulation of dry matter weight per plant in the early stage of soybean growth. In the late stage of soybean growth, dry matter accumulation increased continuously. The soybean yield was the highest when 750 kg/hm^2^ biochar and 42 kg/hm^2^ nitrogen fertilizer were applied. Li et al. conducted a study which demonstrated that under the application of 22.5 kg/hm^2^ nitrogen fertilizer, the addition of 2.4 tons/hm^2^ of biochar significantly enhanced the dry matter accumulation and improved nitrogen utilization efficiency in flue-cured tobacco [49]. Reducing nitrogen fertilizer by 40% compared to the normal supply level is more conducive to promoting the growth and development of pepper fruit and improving the accumulation of quality indicators, leading to better absorption of mineral elements by pepper fruit [50]. In the present study, the highest yield was observed in the B1 and B2 biochar treatments, indicating that optimum biochar application had a specific range, consistent with previous findings [43,45]. The higher yield was associated with the application of high nitrogen fertilizer, with the highest yield observed in the N2B2 and N2B1 combinations.

Numerous studies have investigated the impact of adding biochar on crop quality [19,47,50], as evidenced by various research findings. For instance, biochar could improve the taste of tomatoes by boosting SS values and achieving optimal sugar-to-acid ratios [21]. Additionally, in scenarios involving reduced irrigation, biochar influenced the quality of tomatoes [51]. Moreover, the SS content in vegetables increased with the increase in nitrogen levels, although excessive nitrogen application led to a decrease in sugar content [52]. In our experiment, we observed a gradual increase in SS content with the application of nitrogen fertilizer, consistent with previous research findings. Moreover, an increase in the protein content was observed with the increase in nitrogen application, as nitrogen served as the fundamental building block of amino acids, leading to an increase in protein content within a certain range of nitrogen application [53].

## 5 Conclusions

Utilizing combinations of 25 kg/667 m^2^ nitrogen fertilizer and 0.7% or 1.4% biochar showed promise in boosting plant growth, yield, and fruit quality in pepper cultivation. Optimized blending of biochar and nitrogen fertilizer fostered a conducive growth environment and enhanced the overall quality of pepper cultivation and output. This optimized approach could provide valuable insights for future fertilizer applications in the pepper industry.
